# Infectious Diarrhoea

**Published:** 1915-10

**Authors:** 


**Infectious Diarrhoea.** Carl Ten Broeck and F. C. Norbury, Boston, Boston M. and S. Jour., August 19. 53 cases showed dysentery bacilli. In these, 6 showed also the B. aerogenes capsulatus, the presence of the latter being doubtful in 12, and its absence demonstrated in 35. 37 cases showed no dysentery bacilli. In these, the B. aerogenes capsulatus was present in 7, absent in 19, doubtful in 11. Of 34 cases of indigestion with diarrhoea, the B. capsulatus aerogenes was present in 9, absent in 16, doubtful in 9. Of 14 cases of malnutrition, it was present in 4, absent in 5, doubtful in 5. Of 13 normal cases, it was present in 5, absent in 2, doubtful in 6. These statistics apply to infants. They emphasize the difficulty of finding dysentery bacilli and the fewness of their numbers. They were found, however, in 68 per cent. of 79 cases carefully studied. The Welch bacillus they consider not pathogenic, being more common in mild and normal cases. Likewise, they do not consider B. Proteus vulgaris, Morgan's Bacillus No. 1, para-typhoid bacilli nor B. pyocyaneus as causes of infectious diarrhoea though they may modify the course of an infection started by the dysentery bacillus. II blood cultures and 14 organ cultures in cases of infantile dysentery, were negative with one exception in which the bacilli were found in the blood.

